# *LRP6* β-Propeller Destabilization: Novel Variant, Phenotype and Diagnostic Implications in Tooth Agenesis

**DOI:** 10.1016/j.identj.2025.109367

**Published:** 2026-01-07

**Authors:** Narin Intarak, Tohid Ghasemnejad, Kausar Sadia Fakhruddin, Ayaana Kamal, Sasiprapa Prommanee, Khadijeh Hoda Jahanian, Nigel H. Lovell, Hamid Alinejad-Rokny, Thantrira Porntaveetus

**Affiliations:** a Center of Excellence in Precision Medicine and Digital Health, Department of Physiology, Faculty of Dentistry, Chulalongkorn University, Bangkok, Thailand; b UNSW BioMedical Machine Learning Lab (BML), School of Biomedical Engineering, UNSW Sydney, Sydney, New South Wales, Australia; c Faculty of Dentistry, University of Western Australia, Perth, Australia; d Faculty of Dentistry, University of Saskatchewan, Saskatchewan, Canada; e Faculty of Medicine, Georgian National University SEU, Tbilisi, Georgia; f Clinical Research Center, Faculty of Dentistry, Chulalongkorn University, Bangkok, Thailand; g School of Biomedical Engineering, UNSW Sydney, New South Wales, Australia; h Visiting Scholar (Collaborative Projects), Center of Excellence in Precision Medicine and Digital Health, Department of Physiology, Chulalongkorn University, Bangkok, Thailand; i Clinic of General, Special Care and Geriatric Dentistry, Center for Dental Medicine, University of Zurich, Zurich, Switzerland

**Keywords:** *LRP6*, Oligodontia, Tooth agenesis, Wnt signalling, β-propeller, Healthcare access, Case report

## Abstract

**Background and Objectives:**

Oligodontia, the congenital absence of multiple permanent teeth, is frequently linked to *LRP6* variants. However, the genotype–phenotype correlations remain unclear and non-European cohorts are underrepresented. The objectives of this study were to: (1) characterize the molecular and clinical features of *LRP6* variants identified in unrelated Thai individuals with tooth agenesis; (2) conduct a scoping review of previously published cases; and (3) refine the understanding of *LRP6* genotype–phenotype correlations.

**Materials and Methods:**

A detailed case series analysis of Thai families with congenital tooth agenesis (through exome sequencing and 3D protein modelling) was conducted, and functional validation was performed using computational structural prediction. A reviewed published cases of *LRP6* variants was performed following a PRISMA-ScR-guided scoping review (2005-2025).

**Results:**

p.Asp411Tyr, a novel heterozygous de novo missense change, destabilised the β-propeller domain. While Thai probands expanded the phenotype and genotype spectrum of *LRP6*-associated tooth agenesis, a review of 20 studies showed clustering of variants in β-propeller domains (62%), usually autosomal dominant (78%) but with variable penetrance. The phenotypes ranged from isolated oligodontia to syndromic forms. Thai probands displayed rare ectodermal-associated features (preauricular pits, dry skin) expanding the spectrum.

**Conclusions:**

*LRP6* is a mutational hotspot in tooth development, with variable phenotypical expressivity and penetrance.

## Introduction

Tooth agenesis, the congenital absence of one or more permanent teeth, represents a prevalent developmental anomaly within human dentition. Its clinical spectrum ranges from hypodontia, characterized by the absence of 1 to 5 teeth and affecting 2% to 10% of the population, to the more severe oligodontia, involving the absence of six or more teeth, observed in less than 1% of individuals.^1^^,^^2^ While environmental factors and syndromic conditions contribute to tooth agenesis, a significant proportion of nonsyndromic cases are underpinned by genetic defects that perturb the intricate epithelial-mesenchymal signalling cascades critical for early tooth development.^3^^,^^4^

The canonical Wnt/β-catenin signalling pathway is a pivotal regulator of odontogenesis, orchestrating crucial events in tooth initiation, patterning, and morphogenesis. Low-Density Lipoprotein Receptor-Related Protein 6 (*LRP6*) serves as an indispensable co-receptor for Wnt ligands within this pathway, facilitating downstream signal transduction.^4^^,^^5^ The association between *LRP6* and tooth agenesis was initially established in 2015 by Massink et al,^6^ and subsequent investigations have broadened its implication across a diverse spectrum of both syndromic and nonsyndromic presentations.^7^ Pathogenic variants in *LRP6*, encompassing missense, nonsense, frameshift, and splice-site mutations, frequently localize to the highly conserved YWTD β-propeller domains. These domains are structurally vital for mediating effective ligand-receptor interactions, which are prerequisite for the robust activation of canonical Wnt signaling.^8^, ^9^, ^10^

Disruptions to *LRP6* function frequently compromise the structural integrity and cellular localization of the protein. Common outcomes include protein misfolding, aberrant retention within the endoplasmic reticulum, or diminished cell surface expression, all converging to attenuate canonical Wnt/β-catenin signaling.^11^ Illustratively, the p.Leu344Pro variant has been shown to induce aberrant exon skipping and severe impairment of receptor function, while other reported pathogenic variants, such as p.Arg857His, directly impede LRP6 phosphorylation and subsequent β-catenin accumulation.^12^ These molecular defects manifest as a broad spectrum of clinical phenotypes, ranging from isolated nonsyndromic tooth agenesis to complex syndromic presentations involving skeletal, cardiac, or neurological malformations.^13^^,^^14^

This striking phenotypic variability, even among individuals harbouring identical *LRP6* variants, underscores the complex genetic landscape of tooth agenesis. Such observations strongly suggest mechanisms of incomplete penetrance and highlight the potential influence of genetic modifiers or environmental interactions.^15^ A recent multicentre review by Zhou et al^16^ further emphasized this inherent heterogeneity, advocating for the imperative integration of genetic and functional data to enhance diagnostic precision and prognostic understanding.

However, existing data on *LRP6*-associated tooth agenesis is predominantly derived from European and East Asian cohorts, resulting in a paucity of data regarding other ancestral backgrounds, particularly Southeast Asian populations. To address this disparity and refine the characterization of *LRP6* pathology, this study combines a comprehensive scoping review of published variants with the molecular and clinical analysis of two novel variants identified via exome sequencing in unrelated Thai patients. We aim to elucidate the clinical and functional mechanisms of *LRP6*-mediated tooth agenesis, refine genotype-phenotype correlations, and demonstrate the critical role of functional validation in diverse genetic cohorts.

## Materials and methods

### Participants

Patients presenting with at least one congenitally missing tooth (excluding third molars) were recruited along with their family members from the Dental Hospital of the Faculty of Dentistry, Chulalongkorn University. Individuals presenting with known genetic syndromes or severe systemic malformations were excluded from the study. Physical and orodental examinations of all participants were performed by a senior clinical dental academic (TP). Cephalometric radiography was used for skeletal classification. The signed consent for participation in the study was obtained from all recruits. The study was approved by the Research Ethics Committee of the Faculty of Dentistry, Chulalongkorn University, Bangkok, Thailand (No. 137/2023, HREC-DCU2023-117) per the Declaration of Helsinki (version 2002) and the additional requirement.

### Genetic and bioinformatics analysis

Genomic DNA was isolated from peripheral blood leukocytes.^17^ Exome sequencing was performed at Macrogen Inc. (Seoul, Korea) using a TruSeq Exome Enrichment Kit on the Illumina HiSeq 2000 platform. Variant analysis was conducted using VariantStudio version 3.0.12 (Illumina). The average sequencing depth was 50 ×, with >90% coverage of the target region. Variants were filtered using the following criteria: (1) quality score ≥ 20 and read depth ≥ 10, (2) location in or close to the coding regions, (3) <1% minor allele frequency in the Genome Aggregation Database (gnomAD), 1000 Genomes Project Consortium, dbSNPs, T-Rex^18^ and internal database of 2166 Thai exomes (including at least 1000 healthy individuals), (4) screening against a targeted gene list derived from the Human Phenotype Ontology (HPO) term for ‘Tooth Agenesis’ (HP:0009804).^19^ Finally, the identified variants were classified as ‘pathogenic’ or ‘likely pathogenic’ according to the American College of Medical Genetics and Genomics (ACMG) standard guidelines.^20^^,^^21^ The identified variants were confirmed by sequencing depth analysis. *De novo* mutation was performed using a trio-based approach. The variant was defined as novel if it was not present in the Human Gene Mutation Database (http://www.hgmd.cf.ac.uk/ac/index.php), gnomAD and dbSNPs.

Variant pathogenicity was predicted by the metalearning-based learning rate tuner (MetaLR),^22^ Functional Analysis through Hidden Markov Models (FATHMM),^23^ Rare Exome Variant Ensemble Learner (REVEL)^24^ and AlphaMissense.^25^

The alignment of conserved regions among species was performed by Clustal Omega (version 1.2.4). The LRP6 protein coordinate file was retrieved from the AlphaFold Protein Structure Database (AF-O75581-F1). Structural analysis was performed using The PyMOL Molecular Graphics System, Version 2.0 (Schrödinger, LLC). Mutant residues were modelled *in silico*, and changes in sum partial charge were computed to evaluate electrostatic impact.

### Scoping review

The review followed the PRISMA Extension for Scoping Reviews (PRISMA-ScR) guidelines,^26^^,^^27^ and the case reports were described following the CARE (Case Report) guidelines. We used the PCC (Population, Concept, Context) framework with *Population*: individuals with tooth agenesis hypodontia/oligodontia); *Concept: LRP6* gene mutations/variants; and *Context*: case reports, case series, cohort studies, and genetic studies across syndromic and nonsyndromic forms*.*

*Research question*: What is the current evidence on the clinical and genetic spectrum of nonsyndromic and syndromic tooth agenesis associated with mutations in the *LRP6* gene?

### Eligibility criteria

#### Inclusion criteria

Human studies published between 2005 and April 2025

English-language articles

Case reports, family studies, or cohort studies reporting *LRP6* mutations associated with tooth agenesis (hypodontia/oligodontia)

Functional or expression studies with human-derived mutation data relevant to dental development

#### Exclusion criteria

*In silico*-only studies

*In vitro*-only studies with no clinical/genetic human data

Narrative reviews, editorials, or conference abstracts without original data

Non-English articles and nonhuman studies

### Information sources and search strategy

A literature search was conducted using Google Scholar, PubMed, and Scopus from January 2005 to April 2025, shown in Figure 4. The search strategy combined terms related to the gene of interest, phenotype, and study type as ‘(“LRP6” OR “Low-density lipoprotein receptor-related protein 6”) AND (“tooth agenesis” OR “oligodontia” OR “hypodontia”) AND (“mutation” OR “variant”) AND (“case report” OR “family study” OR “genetic analysis”)’. The reference lists of included articles were manually screened for additional eligible studies. Two independent reviewers (KSF and NI) screened the titles and abstracts, followed by a full-text eligibility assessment. Discrepancies were resolved by consensus.

## Results

### Clinical and genetic findings in two Thai families with LRP6 variants

#### Family 1

##### Patient information

**Fig. 1:**
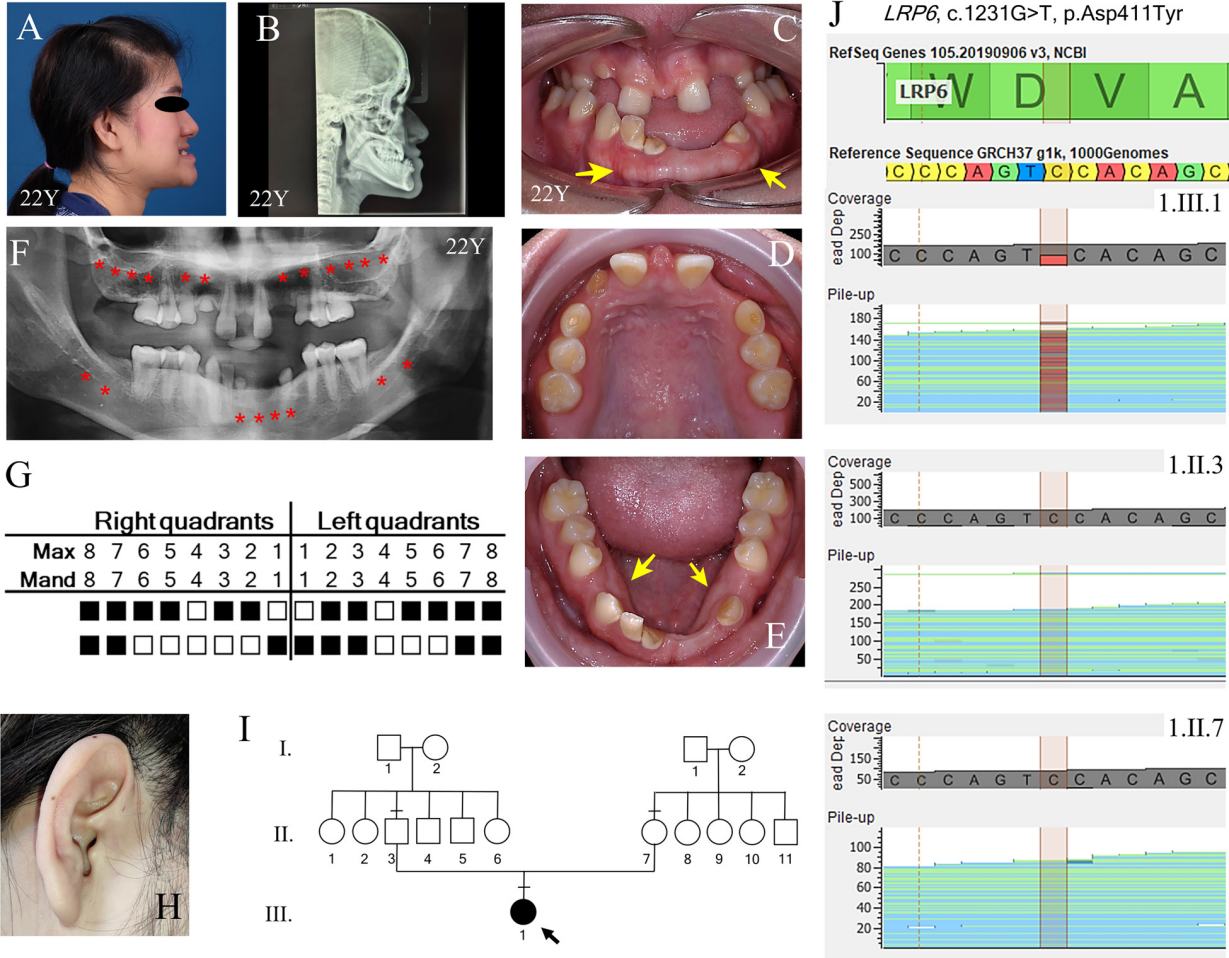
Family 1 phenotype and genetic analysis. A, Lateral face picture demonstrating class 3 skeletal malocclusion. B-C, Intraoral photographs of the proband illustrate the absence of multiple teeth, severe dental caries, retained deciduous teeth, a prominent labial frenum, buccal exostoses (arrows) and, D, Torus mandibularis (arrows). E-F, Panoramic radiograph and tooth agenesis schematic showing the agenesis of a total of 20 permanent teeth (including third molars) (red asterisk), with a taurodontism of the first mandibular molars. G, Proband’s right ear showing preauricular pits. H, Family pedigree illustrating proband (1.III.1) who is a 22-y-old female. The arrow indicates the proband. The black symbol indicates subjects with tooth agenesis. Dash indicates subjects recruited for genetic analysis. I, Sequencing depth chromatograms demonstrate a de novo missense heterozygous novel c.1231G>T (p.Asp411Tyr) variant in *LRP6* in 1.III.1 but not 1.II.3 and 1.II.7.

The proband (1.III.1) was a 22-year-old Thai female born to nonconsanguineous, healthy parents. She was recruited from the dental hospital of the Faculty of Dentistry, Chulalongkorn University, with informed consent obtained under ethical approval. She presented with Class III skeletal malocclusion, anterior open bite, reverse overjet, and oligodontia involving 20 permanent teeth (Figure 1A–E). Intraoral examination revealed retained deciduous teeth, a prominent labial frenum, buccal exostoses (Figure 1C) and torus mandibularis (Figure 1E). The panoramic radiograph confirmed agenesis of teeth 18, 17, 16, 15, 13, 12, 22, 23, 25, 26, 27, 28, 38, 37, 33, 32, 31, 41, 47, and 48, and retention of teeth 55, 53, 63, 65, 71, and 83 (Figure 1G). Hypotaurodontism was observed in the permanent mandibular first molars. Extraoral findings included dry skin, right preauricular pits, and refractive errors (astigmatism and nearsightedness) (Figure 1H). No syndromic features were identified in family members. The patient was counselled and referred for multidisciplinary dental management.

#### Diagnostic assessment

Exome sequencing analysis revealed a novel *de novo* heterozygous missense variant, c.1231G>T (p.Asp411Tyr) in the *LRP6* gene (NM_002336.2, ClinVar accession number: SCV006081039). The variant was classified as ‘likely pathogenic’ according to the ACMG guideline (PS3 PM1 PM2 PP3). The variant was predicted to be deleterious according to the pathogenicity score determined by MetaLR, FATHMM, REVEL, and AlphaMissense (Table 1). Sequencing depth confirmed the presence of c.1231G>T (p.Asp411Tyr) in the proband, but not in the parents. (Figure 1I and J). The position 411 amino acid aspartate (Asp) resides in a β-propeller domain of LRP6 and is highly conserved in various species, including human, chimpanzee, porcine, mouse, bovine, dog, chicken, chameleon, and zebrafish (Figure 3A and B). The LRP6 ectodomain comprises four tandem pairs of YWTD-β-propeller-EGF-like domain (P1E1 to P4E4), followed by three LDLR type A domains.^28^ Each β-propeller is a six-bladed structure that serves as a platform for protein–protein interactions. The substitution of a polar negatively charge Asp to a polar neutral aromatic Tyr resulted in the acquisition of a partial negative charge (with a sum partial charge = −1.4887) (Figure 3C). This apparent conformational change is likely to destabilize the protein structure and attenuate the interaction of various ligands of LRP6.

**Table 1:** An *in silico* pathogenic score and the ACMG classification of the reported variant in this study.

Case	Variant	MetaLR	FATHMM	REVEL	AlphaMissense	ACMG classification
Family 1	c.1231G>T (p.Asp411Tyr)	Deleterious (0.98)	Deleterious	Deleterious (0.97)	Likely pathogenic (0.998)	Likely pathogenic (PS3 PM1 PM2 PP3)
Family 2	c.1252T>C (p.Tyr418His)	Deleterious (0.99)	Deleterious	Deleterious (0.95)	Likely pathogenic (0.989)	Pathogenic (PP3 PS1 PM1 BS1)

#### Family 2

##### Patient information

**Fig. 2:**
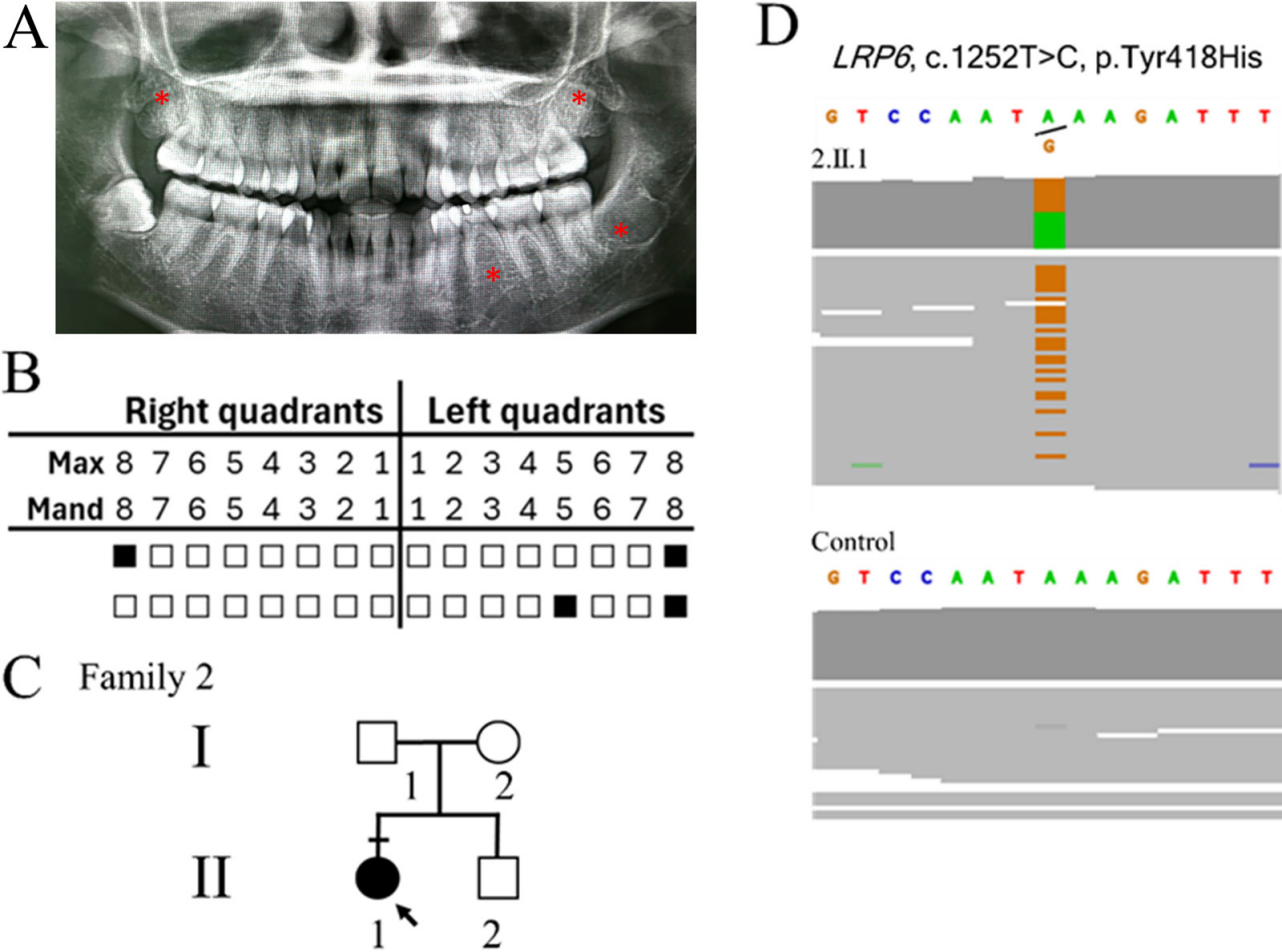
Family 2 phenotype and genetic analysis. A-B, Panoramic radiograph and tooth agenesis schematic showing the agenesis of a total of four permanent teeth (including the third molars) (red asterisk), with the retention of the left primary mandibular molar, and root malformations of the maxillary permanent molars. C, Family pedigree illustrating the proband (2.II.1). The arrow indicates the proband. The black symbol indicates sub-jects with tooth agenesis. Dash indicates subjects recruited for genetic analysis. D, Sequencing depth chromatograms illustrate a missense variant, c.1252T>C (p.Tyr418His) in the *LRP6* gene in 2.II.1 but not unaffected control.

The proband was 30-years old Thai woman (2.II.1) who was born to nonconsanguineous parents, with no reported family history of tooth agenesis. Recruitment and consent followed the same ethical protocol as Family 1. The proband exhibited agenesis of congenital missing permanent teeth, retained deciduous teeth with no observed facial dysmorphic features and ectodermal anomalies. Panoramic radiography confirmed agenesis of teeth 18, 35, 28, 38. retention of teeth 75 (Figure 2A and B). Root malformations of the maxillary permanent molars were observed. No facial dysmorphism or ectodermal anomalies were observed. Her parents and sibling were healthy and had a normal dental history (Figure 2C).

**Fig. 3:**
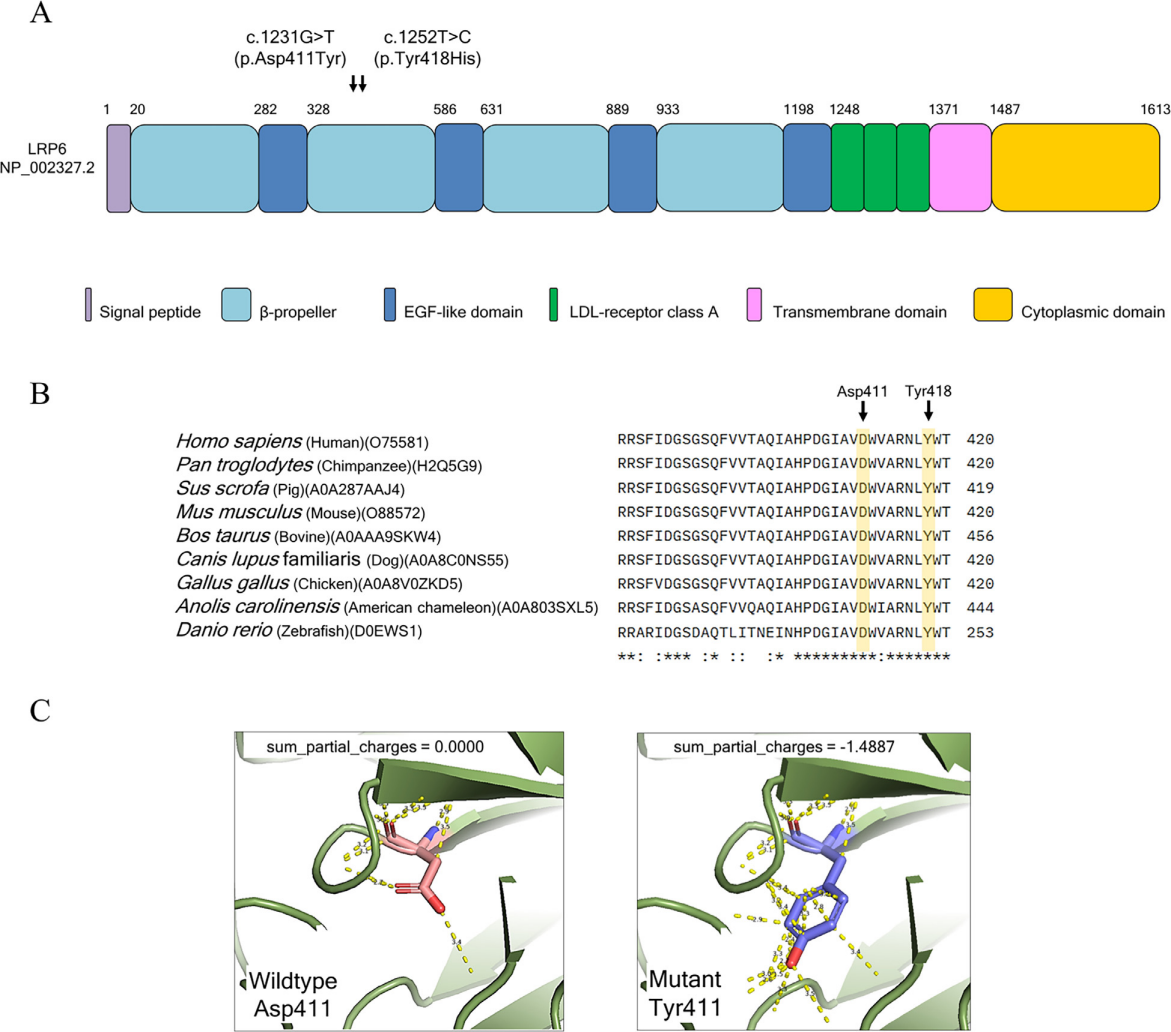
Bioinformatic analysis. A, *LRP6* variants reported in this study. B, The amino acid conservation of LRP6. Asp411, the amino acid affected by c.1231G>T variant, is extremely conserved between species. C, Three-dimensional figures indicate the wildtype Asp411 LRP6 and Tyr411 variant. The dotted yellow lines represent the hydrogen bonds.

**Fig. 4:**
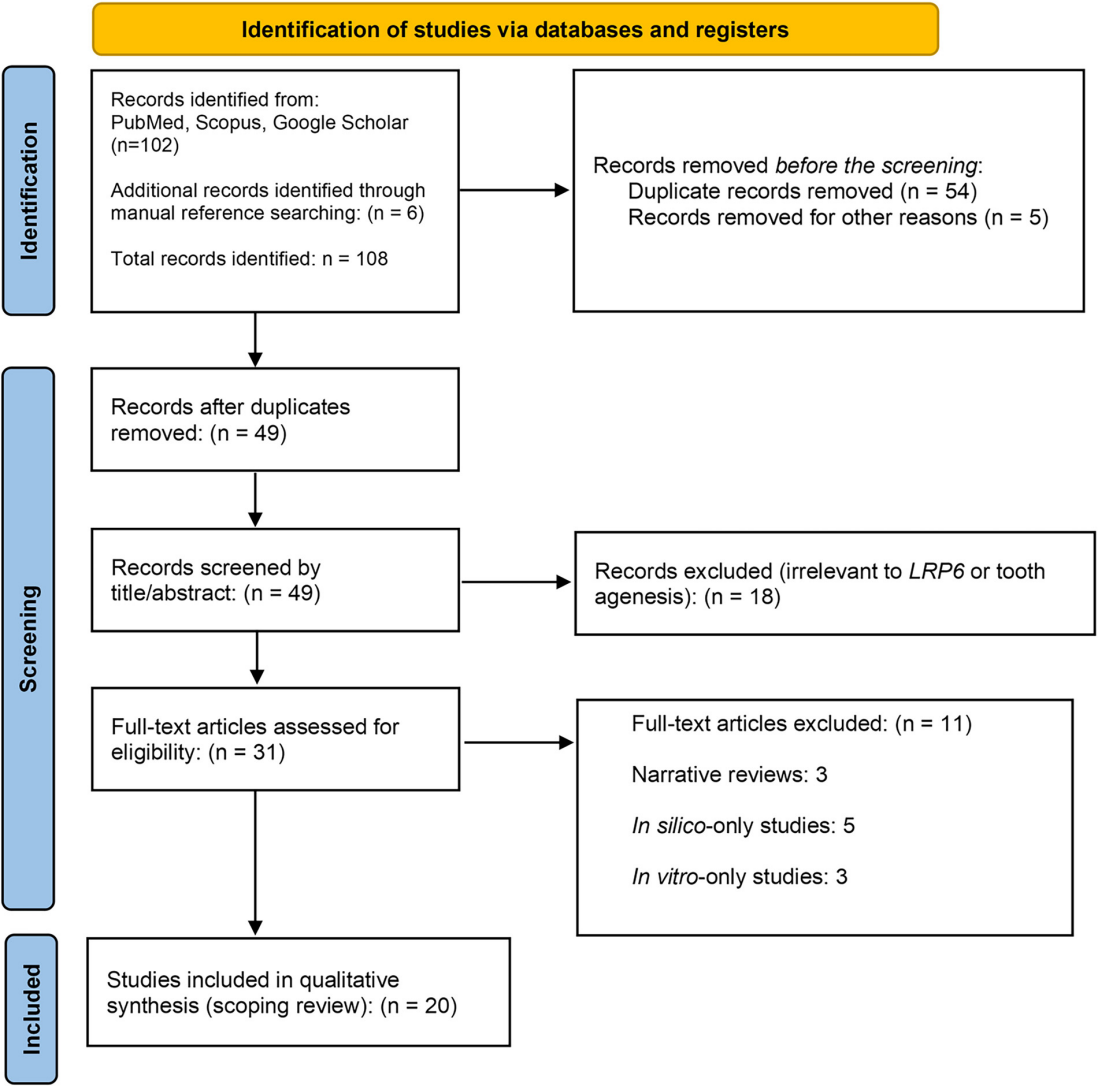
PRISMA flow chart of the literature search and study selection.

#### Diagnostic assessment

Exome sequencing analysis identified a known heterozygous missense variant, c.1252T>C (p.Tyr418His) in *LRP6*. The variant was classified as ‘pathogenic’ according to the ACMG guideline (PP3 PS1 PM1 BS1). The variant was predicted to be deleterious by the pathogenicity score determined by MetaLR, FATHMM, REVEL and AlphaMissense (Table 1). Sequencing depth confirmed the identity of c.1252T>C in the proband but was not in the unaffected control (Figure 2D). Parental samples were unavailable; therefore, the *de novo* status could not be determined. The 418 amino acid tyrosine (Tyr) is located in a conserved structural element in the YWTD β-propeller domains.^29^ This mutation is expected to reduce protein stability since changing Tyr418 to His has been proposed to diminish the hydrogen-bond interaction with the carbonyl backbone of Leu476 on the neighbouring loop that interacts with the EGF domain 2.^15^

## Comparative synthesis of *LRP6* studies with the scoping review

### Demographic, inheritance, and phenotypic features of LRP6 variant carriers with tooth agenesis

There are twenty studies summarized in Table 2 that highlight the association of *LRP6* variants with tooth agenesis, predominantly showing an autosomal dominant inheritance pattern,^8^^,^^13^^,^^15^ though reduced penetrance,^6^ and *de novo* mutations^11^^,^^30^ are also observed. Most cases presented as nonsyndromic oligodontia,^12^^,^^31^^,^^32^ but syndromic forms with features such as orofacial clefts,^7^^,^^33^ ectodermal dysplasia,^8^ or thrombocytopenia^34^ were noted in some families. The clinical phenotype varied, with common patterns including missing maxillary lateral incisors and second premolars.^35^^,^^36^ Additional dental anomalies such as taurodontism,^6^^,^^11^ peg-shaped teeth,^11^^,^^36^ enamel defects,^7^ and microdontia^15^^,^^37^ were frequently reported. Notably, *LRP6*-related tooth agenesis exhibited variable expressivity, even within families,^32^^,^^37^ and could involve digenic interactions, for instance with *WNT10A*.^36^ Functional studies^8^^,^^30^ and 3D modeling^35^^,^^38^ support the role of LRP6 in Wnt signalling disruption, underscoring its critical function in dental development. The data collectively emphasize *LRP6* as a significant gene in both isolated and syndromic tooth agenesis, with implications for genetic counselling and molecular diagnostics.

**Table 2:** Summary of clinical and study-level characteristics included reports on *LRP6* variants associated with tooth agenesis.

Study Year Country Study design	No. of cases	Inheritance pattern	Syndromic? (Y/N)	Clinical phenotype (tooth agenesis pattern)	Other dental features
Present study	2 unrelated individuals with *LRP6* variants	Autosomal dominant (de novo mutation)	Proband 1: No (extraoral findings included dry skin, right preauricular pits, and astigmatism and near sightedness), but not considered syndromic) Proband 2: No	Proband 1: Oligodontia (16 missing permanent teeth excluding third molars), Proband 2: Missing of one permanent tooth,	Proband 1: Hypotaurodontism was observed in the permanent mandibular first molars, buccal exostosis, torus mandibularis. Proband 2: Root malformations of the maxillary permanent molars
Luo et al 2025 China^30^ Case study (2 families) with WES and *in vitro* functional assays	2 probands from 2 unrelated families	*Family 1* (*AXIN2*): Inherited from father *Family 2* (*LRP6*): De novo	No	*Family 1*: 8 missing permanent teeth *Family 2*: 9 missing permanent teeth (excluding third molars)	*Family 1*: Father’s mandibular incisors are abnormal in colour (possible retained or dead pulp); no systemic features
Previdi et al 2024 France^13^ Case report with family pedigree, clinical and radiological characterization, and gene panel sequencing	3 affected individuals (mother and two sons)	Autosomal Dominant (heterozygous *LRP6* variant inherited maternally)	Yes (syndromic with skeletal, dental, cardiac, genital, and neurological anomalies)	All affected individuals exhibited oligodontia (4 permanent teeth); anomalies include hemivertebrae, osteosclerosis, persistent left superior vena cava, cysts, genital and brain malformations	Jaw asymmetry, articulation disorder, small ears, no torus palatinus or exostoses
Kantaputra et al 2022 Thailand^15^ Case series of 14 patients from 8 unrelated families with clinical exams, WES, Sanger sequencing, and protein modelling	14 individuals from 8 families	Autosomal dominant (confirmed segregation in several families)	No (although some had systemic findings like ulcerative colitis, not a defined syndrome)	Tooth agenesis in 5/14 patients; affected teeth included canines, incisors, premolars (variable expression)	Taurodontism, torus palatinus, torus mandibularis, buccal exostoses, mesiodens, odontomas, tooth fusion, microdontia
Keskin et al 2022 Turkey^31^ Cross-sectional cohort study using targeted next-generation sequencing	49 children with nonsyndromic tooth agenesis; 3 individuals with *LRP6* variants	Not specified (sporadic cases; familial segregation not analysed)	No (exclusively nonsyndromic cases included)	Hypodontia involving predominantly second premolars; no oligodontia among *LRP6* variant carriers	Not reported for *LRP6*-specific cases
Lee et al 2022 South Korea^11^ Case report (2 families) with WES, Sanger sequencing, and *in vitro* splicing assay	2 probands (Family 1: female child; Family 2: male adolescent)	*Family 1:* De novo mutation. *Family 2*: Likely autosomal dominant (mother and sister suspected affected)	No	*Family 1*: 16 permanent teeth missing (no primary tooth agenesis); *Family 2*: 17 permanent teeth missing	Both: Taurodontism of first molars. *Family 2*: Peg-shaped lateral incisors
Yue et al 2022 China^12^ Case report with family pedigree, clinical evaluation, WES, and functional validation	2 affected individuals (father and son) from one family	Autosomal Dominant (variant segregated from the affected father to the son)	No (nonsyndromic tooth agenesis)	Proband (male): 6 permanent teeth missing; Father: 5 permanent teeth missing (excluding third molars)	No abnormalities in nails, skin, hair, or sweat glands; normal facial profile
Chu et al 2021 Taiwan (with collaboration from the USA)^36^ Case series (4 unrelated families) with WES, Sanger sequencing, and structural modelling	4 probands with *LRP6* mutations; multiple family members included	Autosomal dominant; 1 compound heterozygote; 2 cases suggest digenic inheritance with WNT10A	No (all cases nonsyndromic)	Oligodontia in all 4 probands (8-14 missing permanent teeth); variable penetrance in family members	Peg-shaped lateral incisors, infraocclusion of primary molars, lobodontia; no taurodontism noted
Goto et al 2021 Japan^39^ Case report with WES and Sanger validation	2 affected individuals (proband and one child); 1 additional carrier (grandfather)	Autosomal dominant	No	Oligodontia (23 missing permanent teeth in proband; primary dentition normal)	None reported
Huang et al 2021 China^32^ Case study with WES, *in silico* modelling, and *in vitro* functional analysis	4 affected individuals from one 4-generation family	Autosomal dominant	No (but two had sparse hair; could indicate ectodermal features, though not formally syndromic)	Variable hypodontia and oligodontia among affected individuals (4-10 missing permanent teeth	None reported beyond sparse hair and dental agenesis.
Wang et al 2021 China^37^ Family-based case study using WES and Sanger sequencing with bioinformatics modelling	3 affected individuals (proband, mother, grandmother)	Autosomal dominant	No (no systemic or ectodermal anomalies identified)	Proband: 17 missing permanent teeth (excluding 3rd molars); Mother and Grandmother: unilateral maxillary lateral incisor agenesis	Microdontia of the remaining lateral incisor (mother and grandmother); no taurodontism noted
Yu et al 2021 China^38^ Cohort study (77 patients with oligodontia) using WES, Sanger sequencing, 3D modelling, and *in vitro* assays	4 unrelated patients with *LRP6* mutations	Mixed: 2 inherited (p.W764* and p.D366Rfs*13); 1 de novo (p.Y66Ifs*4); 1 uncertain (mother unavailable)	Mixed: 1 patient with p.W764* had hypohidrotic ectodermal dysplasia (HED) phenotype; others were nonsyndromic	Oligodontia: 9-18 missing permanent teeth across 4 patients	Cone-shaped maxillary incisors (in 2 cases); no taurodontism reported
Zhang et al 2021 China^35^ Case series (3 unrelated families) with WES, Sanger sequencing, bioinformatics, and 3D protein modelling	7 affected individuals across 3 families	Autosomal Dominant (2 inherited, 1 de novo)	Yes, In Family #704, 2 members had preaxial polydactyly of the hand	Oligodontia (11-16 missing permanent teeth); consistent pattern: maxillary lateral incisor and second premolars are most affected	Retained deciduous teeth, shovel-shaped incisors (IV:1); no taurodontism reported
Zhou et al 2021 China, USA, Turkey, Korea^16^ Systematic review combined with 10 newly recruited family case studies using WES and phenotypic correlation	2 individuals with *LRP6* mutations from the new cohort; 16 cases total with *LRP6* mutations across full dataset	Heterozygous (Dominant); one de novo, others unknown or presumed inherited	No (for the two *LRP6* cases in this study)	*Family 9:* 16 missing permanent teeth *Family 10*: 12 missing permanent teeth	*Family 9*: Not specified *Family 10:* Not specified beyond the number of missing teeth
Brance et al 2020 Argentina (with collaboration from the USA)^40^ Single case report with detailed clinical, radiological, and molecular analysis	One case	Unknown (patient was adopted; biological family history not available)	Yes Presents as a dento-osseous syndrome with HBM and congenital tooth agenesis	Congenital absence of all maxillary and mandibular lateral incisors and one second premolar	Broad jaw and nasal bridge; no torus palatinus or other exostoses
Ross et al 2019 Netherlands^34^ Three-generation case report with genome-wide array analysis	4 individuals from one family	Autosomal dominant	Yes — Oligodontia with concurrent thrombocytopenia due to contiguous gene deletion (*LRP6* + *ETV6*)	Identical tooth agenesis pattern across family: 8-9 missing permanent teeth (eg, upper lateral incisors, lower anteriors)	Taurodontism, dysmorphic ears (underfolded helix), mild thumb hypoplasia
Basha et al 2018 Belgium^33^ Family-based case study within a broader WES cohort (n = 46 families with nsCL/P)	3 carriers in 1 family	Autosomal dominant	No (although cleft and tooth agenesis co-occurred, not classified as syndromic)	Proband: bilateral cleft lip and palate with missing upper lateral incisors; mother: bilateral cleft lip	Tooth agenesis noted; further details on additional dental anomalies not available
Dinckan et al 2018 Turkey (with collaboration from the USA)^46^ Multicentre case series (10 Turkish families) using WES, linkage analysis, array genotyping, and RNA studies	1 family (TF-7) with *LRP6* variant; 3 affected individuals with confirmed segregation	Autosomal dominant	No (mild periocular hyperpigmentation and nasal hypoplasia, but not considered syndromic)	Oligodontia: 16 permanent teeth missing (excluding third molars)	Not specified beyond tooth agenesis
Ockeloen et al 2016 Netherlands, Belgium, Germany, UK, USA^7^ WES and targeted resequencing (MIPs); case series with segregation analysis	2 index patients with detailed data; 5 additional patients with *LRP6* variants (total = 7 with unique variants)	Autosomal Dominant: 1 case had a de novo mutation	Mixed: 1 with TA + orofacial cleft (TA-OFC), others with TA only	Oligodontia: 9-18 permanent teeth missing, some primary teeth missing; one with cleft palate and lip (TA-OFC)	Ankylosis (n = 2), conic/abnormal tooth shape (n = 2), enamel defects (n = 1), impacted and ectopic teeth, malposition
Massink et al 2015 Netherland^6^ Case series with WES and Sanger confirmation	4 unrelated individuals with *LRP6* variants	Autosomal Dominant (with reduced penetrance)	No All cases classified as nonsyndromic oligodontia	Oligodontia (≥6 missing permanent teeth excluding third molars); variable severity and expression	Taurodontism was observed in approximately 1/3 of affected individuals.

TA, tooth agenesis; WES, whole-exome sequencing.

### Molecular and functional characterization of LRP6 variants in nonsyndromic tooth agenesis

The molecular and functional analyses of *LRP6* variants associated with nonsyndromic tooth agenesis reveal diverse pathogenic mechanisms that converge on the disruption of Wnt/β-catenin signalling. As summarized in Table 3, the majority of identified mutations are missense variants located within the β-propeller domains of LRP6, which are critical for ligand-receptor interactions and canonical Wnt pathway activation. Several studies, including those by Dong et al^8^ and Yue et al,^12^ confirmed that such missense mutations, such as p.Cys1032Phe, p.Leu344Pro, destabilize β-propeller structures and impair downstream signalling by reducing LRP6 phosphorylation and nuclear β-catenin accumulation.

**Table 3:** Molecular and functional characteristics of *LRP6* gene variants identified in individuals with nonsyndromic tooth agenesis.

Study	*LRP6* variant (cDNA/protein)	Type of variant	Genetic confirmation method	Functional study performed (Y/N)	Functional effect/pathogenicity evidence	Family history with TA (Y/N)
Present study	c.1231G>T /(p.Asp411Tyr)	Missense (Novel variant)	WES with Sequencing depth validation	Yes (*in silico* modelling, evolutionary conservation, structural analysis)	Pathogenic prediction tools, Variant localizes to the critical first β-propeller; predicted to impair Wnt inhibition	No
Luo et al^30^	c.3074_3082del / p.1025_1028del	Nonframeshift in-frame deletion	WES and Sanger sequencing	Yes	Decreased *LRP6* protein expression, decreased β-catenin levels, and suppression of Wnt/β-catenin signalling (via WB + luciferase assay)	No (*LRP6* mutation confirmed as *de novo* in Family 2)
Previdi et al^13^	c.724T>C / p.Trp242Arg	Missense	Targeted gene panel sequencing + trio/quartet genome sequencing + 3D structural modelling	Yes (*in silico* structural modelling)	Disturbs SOST/DKK1 binding to β-propeller; overactivates Wnt signalling; deleterious by all predictors; affects conserved Trp242 in binding motif	Yes (variant present in mother and both sons; absent in grandmother)
Kantaputra et al^15^	- p.Glu72Lys - p.Lys82Asn - p.Tyr418His - p.Ile773Val - p.Arg32*	4 missense, 1 nonsense	WES + Sanger validation	Yes (*in silico* protein modelling and structural interpretation)	All mutations are located in β-propeller domains; predicted disruption of WNT ligand binding or glycosylation; Arg32* leads to complete loss of function	Yes, present in several multigenerational families; segregated with phenotype
Keskin et al^31^	c.3388G>A / p.Asp1130Asn c.3076C>T / p.Arg1026Cys c.1603A>T / p.Ile535Leu	Missense	Targeted NGS panel + *in silico* prediction	No (only *in silico* tools like MutationTaster, SIFT, DANN used)	Predicted uncertain significance; possibly disturbing functional domains or protein structure	No family history or segregation data available
Lee et al^11^	*Family 1:* c.1870dupA / p.Met624Asnfs*29 (frameshift) *Family 2:* c.1762+2T>C (splice site)	*Family 1:* Frameshift *Family 2:* Splice-site mutation leading to exon skipping and frameshift	WES + Sanger sequencing	Yes (minigene assay for splicing + in silico modelling)	Exon 8 skipping confirmed *in vitro* (c.1762+2T>C); Both mutations introduce premature stop codons → mRNA decay	*Family 1:* No (confirmed *de novo* by paternity test) *Family 2:* Yes (suspected affected mother and sister)
Yue et al^12^	c.1031T>C / p.Leu344Pro	Missense	WES + Sanger sequencing + segregation analysis	Yes	Impaired Wnt/β-catenin signalling; reduced nuclear β-catenin accumulation; variant located in YWTD β-propeller domain	Yes (father and son both affected; variant segregates)
Chu et al^36^	c.3754C>T / p.Gln1252* c.503T>G / p.Met168Arg c.2260G>C / p.Ala754Pro c.3224A>G / p.Asn1075Ser	1 nonsense, 3 missense (2 novel, 1 likely pathogenic)	WES + Sanger validation + segregation analysis	Yes (structural destabilization via ∆∆G calculations and conservation analysis)	Missense mutations (eg, p.Met168Arg, p.Ala754Pro) predicted to be destabilizing; digenic inheritance suggested in 2 cases	Yes in 3/4 families; 1 compound heterozygote with inheritance from both parents
Goto et al^39^	c.1924dup / p.Ile642Asnfs*11	Frameshift (resulting in a premature stop codon and a truncated extracellular protein)	WES + Sanger sequencing	No	Predicted loss of transmembrane and cytoplasmic domains; not present in population databases; protein truncation	Yes
Huang et al^32^	c.2570G>A / p.Arg857His	Missense	WES + Sanger sequencing	Yes	Impaired *LRP6* maturation and phosphorylation; disrupted Wnt signalling; structural instability in MD simulations	Yes (segregated in 6 family members, 4 of whom were phenotypically affected)
Wang et al^37^	c.711G>T / p.Leu237Phe	Missense	WES + Sanger validation	Yes (bioinformatics analysis and 3D structural modelling)	Highly conserved site: predicted to be deleterious by multiple tools (SIFT, PolyPhen-2, MutationTaster, CADD); altered 3D structure; hydrogen bond loss	Yes (segregation confirmed across three generations)
Yu et al^38^	c.2292G>A / p.Trp764* (nonsense) c.195dup / p.Tyr66Ilefs4 (frameshift) <br> - c.1095dup / p.Asp366Argfs*13 (frameshift) c.1681C>T / p.Arg561* (nonsense)	2 nonsense and 2 frameshift (all truncating, loss-of-function)	WES + Sanger sequencing + segregation analysis	Yes	All 4 mutants truncated the protein; none were secreted; all suppressed Wnt/β-catenin activity; dominant-negative effect	Yes for 2 (families #46 and #26); No for 1 (*de novo*); Unknown for 1 (mother deceased)
Zhang et al^35^	c.2840T>C / p.Met947Thr (Family #704) c.1154G>C / p.Arg385Pro (Family #221) c.1406C>T / p.Pro469Leu (Family #227)	All 3 are missense mutations	WES + Sanger validation + familial co-segregation	Yes (*in silico* modelling, evolutionary conservation, structural analysis)	Predicted damaging by multiple tools (SIFT, PolyPhen-2, PROVEAN, MutationTaster); conformational disruption of EGF-like repeats in *LRP6*	Yes, for Families #704 and #221; No (*de novo*) for Family #227
Zhou et al^16^	*Family 9*: c.1003C>T / p.Arg335* Family 10: c.2747G>T / p.Cys916Phe	*Family 9:* Nonsense (truncating) *Family 10:* Missense	WES + Sanger sequencing	No (functional predictions only via in silico tools like SIFT, PolyPhen-2)	Predicted to be pathogenic; not present in gnomAD; damaging scores in SIFT/PolyPhen-2	Not detailed; family pedigrees not shown for these two *LRP6* cases
Brance et al^40^	c.678T>Adel679-684 / p.His226Gln-del227-228ProPhe	Complex indel: missense + 2-amino acid in-frame deletion (nonframeshift) in the first β-propeller domain	Targeted NGS panel + Sanger sequencing + cloning of exon 4	No direct functional assay; pathogenicity inferred from location and protein modelling	Variant localizes to the critical first β-propeller; predicted to impair Wnt inhibition and cause HBM phenotype	Unknown (adopted patient)
Ross et al^34^	290 kb contiguous gene deletion in 12p13.2 (deletes exons 16-23 of *LRP6*)	Deletion (large interstitial deletion, structural variant)	Genome-wide array (Affymetrix CytoScan HD array) with family validation	No direct functional study: functional loss inferred based on gene dosage and deleted exons	Loss of *LRP6* exons 16-23 likely truncates the Wnt receptor; phenotype matches prior *LRP6* loss-of-function cases	Yes, consistent segregation across three generations
Basha et al^33^	c.3373C>T / p.Arg1125*	Nonsense (truncating) mutation	WES + Sanger validation	No (no *in vitro* or *in silico* assays performed; variant located in conserved YWTD repeat domain)	Variant absent from gnomAD; located in the critical fourth β-propeller domain of *LRP6* (likely loss-of-function)	Yes (proband, mother affected; brother was an unaffected carrier)
Dinckan et al^46^	c.3607+3_6del / p.?	Splice-site variant (intronic deletion near exon 16)	WES, Sanger sequencing, qRT-PCR	Yes	Quantitative PCR showed reduced *LRP6* mRNA expression, indicating nonsense-mediated decay	Yes (segregation in affected members, absent in unaffected relatives)
Ockeloen et al^7^	c.4594delG (p.Cys1532fs) [frameshift] c.3398-2A>C (splice site) + 5 more: 2 splice, 3 missense	Frameshift, canonical splice-site, missense (all rare and likely pathogenic)	WES and MIP-based targeted resequencing, confirmed by Sanger sequencing	Partially – yes (mouse model immuno-histochemistry and Trp63 regulation assays)	Expression in enamel epithelium; variant p.Cys1532fs causes protein truncation and likely disrupts Wnt signalling	Yes, For most confirmed segregation in 6/7 families; 1 *de novo* mutation (TA2)
Massink et al^6^	c.56C>T / p.Ala19Val (missense) c.1144_1145dupAG / p.Ala383Glyfs*8 (frameshift) <br> - c.1779dupT / p.Glu594* (nonsense) c.2224_2225dupTT / p.Leu742Phefs*7 (frameshift)	Missense, nonsense, and frameshift (all predicted loss-of-function)	WES followed by Sanger sequencing and segregation analysis	Yes (only for c.56C>T / p.Ala19Val)	Abrogated Wnt signalling; p.Ala19Val caused ER retention and failed glycosylation; no cell surface localization or Wnt activation	Yes All four individuals had family members with tooth agenesis

DANN, deep artificial neural network; DKK1, Dickkopf-related protein 1; NMD, nonsense-mediated decay; SIFT, sorting intolerant from tolerant; SOST, sclerostin; WB, Western blot; WES, whole-exome sequencing.

*In silico* and *in vitro* functional studies, including those by Previdi et al^13^ and Huang et al,^32^ further revealed that variants such as p.Trp242Arg and p.Arg857His, disrupt critical ligand-binding motifs and protein maturation, respectively, leading to overactivation or suppression of Wnt signalling. Similarly, Wang et al^37^ demonstrated through structural modelling that the p.Leu237Phe variant alters hydrogen bonding networks, further underscoring the importance of conformational integrity for LRP6 function. Other mutations, such as those reported by Keskin et al^31^ for example, p.Asp1130Asn, p.Arg1026Cys, were predicted to affect interdomain stability but lacked direct functional validation.

Nonmissense variants, including frameshift and splice-site mutations, have also been characterized. For instance, Lee et al^11^ and Goto et al^39^ identified frameshift mutations, such as p.Met624Asnfs29 and p.Ile642Asnfs11, that result in truncated proteins and predicted loss-of-function due to nonsense-mediated decay or disruption of cytoplasmic domains. Splice-site variants, c.1762+2T>C,^11^ were shown via minigene assays to cause exon skipping, leading to frameshifts and altered *LRP6* transcripts, thereby compromising functional protein output.

Additionally, compound heterozygous and complex indel mutations, such as those reported by Chu et al^36^ and Brance et al,^40^ indicates that multilocus or digenic interactions may underlie some cases of tooth agenesis. The presence of *de novo* mutations and variable inheritance patterns, including autosomal dominant transmission and incomplete penetrance, as shown by Ross et al,^34^ and Zhang et al,^35^ further reflects the genetic complexity of *LRP6*-related phenotypes. Collectively, these findings underscore the centrality of LRP6 structural domains in maintaining Wnt pathway fidelity and tooth morphogenesis.

## Discussion

The identification of a novel *de novo* missense variant, c.1231G>T (p.Asp411Tyr) and known missense variant, c.1252T>C (p.Tyr418His) in *LRP6* within Thai individuals diagnosed with oligodontia substantially reinforces the indispensable role of *LRP6* in orchestrating human dental development. Our findings not only corroborate but also significantly expand upon previous reports of *LRP6*-associated tooth agenesis, reaffirming the gene’s multifaceted contribution to both nonsyndromic and syndromic forms of oligodontia, often mediated by diverse pathogenic mechanisms.^41^ The subsequent discussion aims to rigorously contextualize these findings within the existing scientific literature, emphasizing key genetic, phenotypic, and mechanistic consistencies, while also critically addressing the inherent limitations of the current study.

### Phenotypic spectrum: from isolated oligodontia pleiotropic ectodermal manifestations

While comprehensively detailed in the Results section, we underscore pertinent phenotypic features here to facilitate a robust contextualization of our *LRP6* findings. Both probands presented with tooth agenesis, manifesting as the absence of 20 and 4 permanent teeth, respectively – a pattern highly consistent with previously documented *LRP6* pathogenic variants. The co-occurrence of additional dental anomalies, such as taurodontism, peg-shaped incisors, exostosis, torus mandibularis and molar root malformation serves as compelling evidence of *LRP6’s* pleiotropic influence extending beyond tooth number to encompass intricate tooth morphology. Furthermore, the observation of subtle ectodermal-associated signs in one proband, specifically preauricular pits and dry skin, parallels features reported in rare syndromic *LRP6* cases, including those with oligodontia and orofacial clefts, eg, as discussed by Previdi et al^13^ who described *LRP6* variants associated with broader systemic involvement. Conversely, other studies, such as that by Yue et al,^12^ have reported *LRP6* variants leading to isolated oligodontia devoid of extra-dental ectodermal features. This striking dichotomy vividly illustrates the marked phenotypic heterogeneity, even among individuals carrying identical or functionally analogous *LRP6* variants.

Collectively, these observations provide compelling evidence for significant variable expressivity in *LRP6*-related conditions. This clinical complexity strongly implicates the potential for digenic interactions, particularly with genes involved in parallel or convergent signalling pathways such as WNT10A,^42^ or the influence of other genetic or environmental modifiers that have not yet been identified.^43^

### Pathogenic mechanisms: disrupting Wnt/β-catenin signalling

Pathogenic missense variants within the highly conserved YWTD β-propeller domains of LRP6 are well-established causes of disrupted Wnt ligand binding and subsequent signalling impairment. In the present study, both p.Asp411Tyr and p.Tyr418His substitutions are located at highly conserved residues within the critical β-propeller domain. Despite their distinct molecular nature, our structural modelling suggests a functional convergence toward protein destabilization. The p.Asp411Tyr variant is predicted to profoundly alter the electrostatic surface potential, leading to a loss of crucial hydrogen bonding networks. Similarly, the p.Tyr418His substitution is expected to compromise stability by diminishing the hydrogen-bond interaction with the carbonyl backbone of Leu476 on the neighbouring loop. Thus, both variants likely impair the structural integrity required for effective LRP6-ligand interaction.^15^ Such structural compromises are anticipated to directly impair effective Wnt ligand-receptor engagement and downstream signal transduction. These i*n silico* predictions are strongly congruent with established literature on other *LRP6* variants, such as p.Arg857His and p.Leu237Phe, which have been experimentally shown to either diminish receptor phosphorylation or destabilize the overall β-propeller fold. The severe dental phenotype observed in our proband further corroborates a definitive structure-function relationship, emphasizing that even subtle single amino acid substitutions within these critical domains can profoundly compromise canonical Wnt signalling and precipitate a wide spectrum of dental anomalies.

### Predicted structural impact of LRP6 variants

In a recent study*,* the *in silico*-predicted structural perturbations associated with the Thai p.Asp411Tyr variant find compelling corroboration in experimental functional data derived from other studies. For instance, Huang et al^32^ meticulously demonstrated that mutant *LRP6* constructs exhibit impaired post-translational modifications, specifically evidenced by reduced glycosylation (manifesting as immature protein bands) and significantly attenuated phosphorylation upon Wnt3a stimulation. These findings unequivocally confirm a profound disruption of the canonical Wnt signalling cascade at multiple levels. Complementarily, investigations by Massink et al^6^ and Ockeloen et al^7^ revealed that pathogenic *LRP6* variants, including p.Ala19Val and p.Arg173Gly, induced aberrant endoplasmic reticulum retention and markedly reduced cell surface membrane localization. This mistrafficking ultimately attenuates β-catenin signalling by precluding proper ligand interaction at the cell surface. Collectively, these mechanistic insights highlight how diverse *LRP6* variants, through structural compromise and impaired cellular processing, converge to dysregulate the Wnt/β-catenin pathway, leading to the observed developmental defects.

### Phenotypic correlations and clinical implications

The phenotypic presentation of our Thai proband harbouring the novel p.Asp411Tyr variant, characterized by severe oligodontia (20 missing teeth) and concomitant ectodermal features such as dry skin, aligns notably with the spectrum of previously described syndromic *LRP6* cases. This consistency is exemplified by reports such as Ross et al,^34^ who documented oligodontia alongside broader systemic features attributed to contiguous gene deletions involving *LRP6*. Similarly, Huang et al^32^ observed *LRP6* individuals carrying the p.Arg857His variant to exhibit variable expressivity, with tooth agenesis ranging from 4 to 10 missing teeth, often accompanied by ectodermal manifestations like sparse hair. Furthermore, Wang et al^37^ reported the p.Leu237Phe variant in a Chinese family, noting a recurrent pattern of missing lateral incisors and premolars – phenotypes that strikingly parallel our current observations. These comparative analyses underscore a consistent genotype-phenotype landscape, where *LRP6* defects can manifest with diverse but recognizable patterns of dental and ectodermal involvement.

Collectively, the accumulating evidence, including our present findings, robustly supports the conclusion that pathogenic missense variants located within the critical YWTD β-propeller domains (eg, p.Asp411Tyr, Tyr418His, p.Arg857His, p.Leu237Phe) primarily exert their effect by destabilizing LRP6 protein structure, thus abrogating effective Wnt ligand binding, and consequently precipitating profound downstream Wnt/β-catenin signalling deficits. These molecular disruptions consistently manifest clinically as severe oligodontia, frequently co-occurring with various ectodermal anomalies,^44^^,^^45^ thereby unequivocally underscoring LRP6’s indispensable and dual role in both dental and broader ectodermal development. While *in silico* predictions and *in vitro* evidence, including our own, strongly support the pathogenicity of these variants, the ultimate confirmation of functional consequences *in vivo* necessitates further rigorous validation using patient-derived cellular models, for instance, through Wnt reporter assays in relevant cell lines such as HEK293T cells. Based on the consistent body of findings across diverse populations, we advocate for the prioritization of *LRP6* variant screening, specifically targeting the β-propeller domains, in patients presenting with severe oligodontia, particularly when accompanied by suggestive ectodermal-associated features. Beyond improving diagnostic precision, this proactive approach holds immediate translational value for comprehensive treatment planning. Early identification allows clinicians to anticipate progressive phenotypes, such as maxillary hypoplasia or xerostomia, enabling proactive preservation of the alveolar ridge. This molecular insight is pivotal for sequencing orthodontic and prosthetic interventions. For instance, in patients genetically predisposed to Class III malocclusion, early interceptive orthodontics can be utilized to modify skeletal growth, whereas the placement of dental implants must be postponed until the cessation of vertical craniofacial growth. Since osseointegrated fixtures act as ankylosed units, premature placement risks infra-occlusion. Thus, genetic verification serves as a vital decision-support tool to optimize orthodontic outcomes, minimize surgical invasiveness, and maximize the longevity of prosthetic rehabilitation.

### Study limitations

While this investigation offers significant insights into *LRP6*-associated tooth agenesis, it is imperative to acknowledge several inherent limitations. Firstly, the assessment of pathogenicity for the novel p.Asp411Tyr and a known Tyr418His variant predominantly relied on *in silico* prediction tools and computational structural modelling. Although highly informative, these approaches provide correlative evidence. Therefore, direct experimental functional assays, such as robust Wnt/β-catenin signalling reporter analyses conducted in patient-derived cellular models or CRISPR-edited cell lines, are unequivocally required to definitively validate the precise mechanistic impact of this variant. Secondly, our study, like many rare disease investigations, is limited by the sample size of affected individuals. While our findings are consistent with established literature, a larger cohort would strengthen statistical power and allow for more robust genotype-phenotype correlation analyses.

### Clinical and diagnostic implications

The integration of granular phenotypic characterization with comprehensive genetic analysis, as presented in this study, significantly contributes to a more refined and nuanced understanding of *LRP6*-related oligodontia. The robust evidence herein unequivocally supports prioritizing *LRP6* genetic screening in patients exhibiting severe tooth agenesis, particularly when such presentations are compounded by additional dental anomalies (eg, taurodontism) or suggestive ectodermal signs. However, given the well-documented variable penetrance and expressivity observed in *LRP6*-related conditions, clinicians must exercise judicious caution in interpreting genotype-phenotype correlations. It is crucial to provide comprehensive genetic counselling that thoroughly addresses the potential for variable expressivity, even among asymptomatic carriers, to manage expectations and inform reproductive planning.

Furthermore, to enhance interstudy comparability and fortify the precision of future genotype-phenotype correlations, we strongly advocate for the standardized application of Human Phenotype Ontology (HPO) terms for all dental and ectodermal-associated traits. Such standardization is critical for advancing the collective understanding and diagnostic accuracy of this complex genetic disorder.

## Conclusion

*LRP6* variants cause nonsyndromic tooth agenesis in Thai patients by destabilizing the β-propeller and disrupting Wnt signalling. This reinforces LRP6’s role in ectodermal development. Pathogenic variants cluster in the β-propeller, leading to autosomal dominant inheritance with variable severity (from NSTA to syndromic ectodermal anomalies). Future research requires functional validation and population-specific screening to enhance precision diagnosis and counselling.

## Patient consent statement

Informed consent was obtained from all participants involved in this study, encompassing approval for both the publication of data and photographs.

## Author contributions

The study was conceived and designed by T.P., N.I. and H.A.R. All data analysis including gene and variant curation, annotation, Scopic reviewing were performed by N.I., K.S.F. and A.K. with the supervision of H.A.R. and T.P. Data collection and data preparation were carried out by N.I., S.P., T.G., and K.J. Figures and tables were produced by N.I. and A.K. The manuscript draughting was written by N.I and K.S.F. Manuscript was critically edited by T.P., N.I., NL and H.A.R. All authors have read and approved the final version of the paper.

## Funding

HAR was supported by the UNSW Scientia Program Fellowship and the Australian Research Council Discovery Early Career Researcher Award (DECRA), under grant DE220101210. HAR was also supported by the Second Century Fund (C2F), Chulalongkorn University, as a Visiting Scholar for collaborative projects involving data collection, data processing, and APC support. TP was supported by the Ratchadaphiseksomphot Endowment Fund, Chulalongkorn University (The Exchange Faculty Travel Grant; Grant No. CTG168027), Health Systems Research Institute (68-032, 68-059), Faculty of Dentistry (DRF69_015), Thailand Science Research and Innovation Fund Chulalongkorn University (HEA_FF_69_036_3200_003).

## Conflict of interest

The author is an Editorial Board Member/Editor-in-Chief/Associate Editor/Guest Editor for this journal and was not involved in the editorial review or the decision to publish this article.
